# One-step surgery with multipotent stem cells and Hyaluronan-based scaffold for the treatment of full-thickness chondral defects of the knee in patients older than 45 years

**DOI:** 10.1007/s00167-016-3984-6

**Published:** 2016-01-14

**Authors:** Alberto Gobbi, Celeste Scotti, Georgios Karnatzikos, Abhishek Mudhigere, Marc Castro, Giuseppe M. Peretti

**Affiliations:** 1 Orthopaedic Arthroscopic Surgery International (O.A.S.I.) Bioresearch Foundation, Gobbi Onlus, Via GA Amadeo 34, 20133 Milan, Italy; 2 IRCCS Istituto Ortopedico Galeazzi, Milan, Italy; 3 Philippine Orthopeadic Institute, Makati Medical Center, Makati, Philippines; 4 Department of Biomedical Sciences for Health, University of Milan, Milan, Italy

**Keywords:** Bone Marrow Aspirate Concentrate (BMAC), Mesenchymal Stem cells (MSCs), Hyaluronan, Scaffold, Cartilage, Cartilage lesion

## Abstract

**Purpose:**

The aim of this study is to prospectively evaluate the medium-term effectiveness and regenerative capability of autologous adult mesenchymal stem cells, harvested as bone marrow aspirate concentrate (BMAC), along with a hyaluronan-based scaffold (Hyalofast) in the treatment of ICRS grade 4 chondral lesions of the knee joint, in patients older than 45 years.

**Methods:**

A study group of 20 patients with an age >45 years (mean 50.0 ± 4.1 years) was compared to a control group of 20 patients with an age <45 years (mean 36.6 ± 5.0). Patients were prospectively evaluated for 4 years. All patients were evaluated with MRI, KOOS, IKDC, VAS and Tegner scores preoperatively and at two-year and final follow-up.

**Results:**

At final follow-up, all scores significantly improved (*P* < 0.001) as follows: all KOOS score categories; Tegner 2 (range 0–4) to 6 (range 4–8) and 3 (range 0–6) to 6 (range 3–10); IKDC subjective (39.2 ± 16.5 to 82.2 ± 8.9) and (40.8 ± 13.9 to 79.4 ± 14.6), in the study and control group respectively. In addition, we show that results are affected by lesion size and number but not from concomitant surgical procedures. MRI showed complete filling in 80 % of patients in the study group and 71 % of patients in the control group. Histological analysis conducted in three patients from the study and two patients from the control group revealed good tissue repair with a variable amount of hyaline-like tissue.

**Conclusion:**

Treatment of cartilage lesions with BMAC and Hyalofast is a viable and effective option that is mainly affected by lesion size and number and not by age. In particular, it allows to address the >45 years population with functional outcomes that are comparable to younger patients at final follow-up.

**Level of evidence:**

Prospective cohort study, Level II.

## Introduction

The treatment of cartilage defects is a current clinical challenge. Two retrospective reviews of knee arthroscopies demonstrated an underestimated incidence of these lesions [7, 34]. In particular, a 60 % incidence of chondral lesions has been demonstrated in all patients aged between 40 and 50 years undergoing a knee arthroscopy, as well as an increase in symptoms and disability with age [7, 34]. When an injury to the articular cartilage occurs, the resulting reparative fibrocartilage has inferior biological and biomechanical properties, compared to native hyaline cartilage, and may undergo degenerative changes ultimately leading to osteoarthritis (OA) [15, 23]. For this reason, regenerating the articular surface is of paramount importance.

Several surgical techniques for the regeneration of the articular cartilage have been proposed. Among them, two-step autologous chondrocyte implantation (ACI) procedures have been shown to provide good results, promoting formation of new hyaline-like cartilage tissue [3, 14, 21], while other techniques, such as microfracture, do not result in a hyaline and durable repair tissue [10]. In particular, hyaluronic Acid (HA), a natural polymer present in the majority of soft tissues, has been used as a scaffold for ACI with good clinical and histological results, promoting formation of new hyaline-like cartilage tissue [3, 14, 21]. However, despite the satisfactory clinical results reported and the potential for delaying development of OA [8], the cost-effectiveness of these staged procedures has been questioned. Drawbacks affecting the reproducibility and quality of clinical outcomes include the following: (1) limited chondrocytes redifferentiation upon culture in vitro [4], (2) reduced chondrogenic potential of chondrocytes in aged patients [1] and (3) donor site morbidity [24].

In this regard, the use of bone marrow aspirate concentrate (BMAC), containing mesenchymal stem cells (MSCs), coupled to a HA-based scaffold recently emerged, based on previous experience with ACI. This technique has the potential to overcome typical limitations of cell-based two-step procedures, representing a promising one-step option to repair cartilage defects due to the chondrogenic potential and easy availability of MSC. In particular, early clinical experience with BMAC and HYAFF^®^11 (Hyalofast, Anika Therapeutics Inc. Massachusetts, USA), a hyaluronan-based scaffold, resulted in satisfactory short-term clinical results and tissue repair, thus prompting for further studies with longer follow-up or with broader indications [11–13]. Additionally, the specific ability of MSCs on the HYAFF^®^11 scaffold to differentiate into chondrogenic cells was demonstrated by the expression and production of specific extracellular matrix molecules [5, 9, 16, 22, 28]. However, a decrease in number and chondrogenic potency of MSCs in aged patients with also a decreased differentiation, proliferation and self-renewal potential, has been reported in vitro [2]. Still, there is neither clinical nor pre-clinical evidence for a reduced cartilage repair potential of MSCs in aged patients to date.

The aim of this work is to prospectively assess the impact of age and of associated morbidities in the >45 years population on the treatment of ICRS grade 4 cartilage defects with BMAC and HYAFF^®^11.

## Materials and methods

A study group of 20 patients aged >45 years (mean age 50.0 ± 4.1 years) and a control group of 20 patients aged <45 years (mean age 36.6 ± 5.0 years) with full thickness cartilage lesions of the knee joint underwent cartilage implantation using BMAC soaked HA scaffold. All patients were prospectively evaluated for 4 years (study group: 48.7 ± 12.6 months; control group: 52.3 ± 12.2 months). Demographic data are summarized in Table 1.

**Table 1:** Demographic data

Demographic data	Study group		Control group	
No. of patients	20		20	
Age (years)	50 ± 4		36.6 ± 5	
Average lesion size (cm^2^)	8.5 ± 5.9		9.8 ± 4.4	
Size of lesion	Lesion <8 cm^2^	Lesion >8 cm^2^	Lesion <8 cm^2^	Lesion >8 cm^2^
	11	9	9	11
Number of lesion	Single lesions	Multiple lesions	Single lesions	Multiple lesions
	12	8	16	4
Concomitant procedures	Single surgery	Multiple surgeries	Single surgery	Multiple surgeries
	6	14	8	12

Values are described as mean ± SD

Inclusion criteria were as follows: age between 45 and 60 years for the study group and 20–44 years for the control group; BMI between 20 and 30 kg/m^2^; full thickness ICRS grade 4 cartilage lesions with size ≥4 cm^2^; and clinical symptoms of pain, swelling, locking or giving way. Co-existing knee pathologies such as tibiofemoral axial malalignment, patellofemoral maltracking and ligamentous insufficiency were treated during the same surgical procedure (Table 2). Exclusion criteria were as follows: deep osteochondral lesions requiring bone grafting; Kellgren and Lawrence grade ≥2, tricompartmental OA, osteonecrosis and inflammatory arthropathy; patients with other general medical conditions (e.g. diabetes mellitus, rheumatoid arthritis, etc.); multiple and recent (<3 months) intra-articular injections with steroids; deformity or OA at ipsilateral and contralateral hip or ankle joints; or possible non-compliance to the proposed rehabilitation protocol. All patients gave informed consent prior to their inclusion in the study and all the procedures were performed by the senior author.

**Table 2:** Concomitant procedures

Concomitant lesion	Study group	Control group	Concomitant procedure
Femoro-tibial malalignment	9	2	High tibial medial opening wedge osteotomy
ACL tear	3	1	ACL reconstruction
Patellar maltracking	1	3	Lateral release
	1	5	Fulkerson procedure
Meniscal lesion	0	1	Meniscectomy

Functional evaluation was performed by visual analogue scale (VAS) for pain (0 = no pain, 10 = worst pain), International Knee Documentation Committee (IKDC) [17, 32], Knee injury & Osteoarthritis Outcome Score (KOOS) [29] and Tegner [32], and mean measurements were adjusted to one decimal. Scores were obtained preoperatively, at two-years and at final follow-up. Radiographic and magnetic resonance imaging (MRI) results were collected preoperatively, at two-years and final follow-up. Standard radiographic evaluation included standing antero-posterior (AP) long-leg views—including hips and ankles—standing AP/lateral views of the knee, skyline patellofemoral views and standing views with the knee bent at 45°. MRI protocols were not standardized since the MRI scans were performed at different facilities. Features of the graft that were assessed included the following: the extent of filling of the defect by repair tissue; integration of the graft to the native cartilage and to the subchondral bone, hypertrophy of the graft, and presence of subchondral oedema or cysts. All patients followed the same four-phase rehabilitation protocol [20].

### Surgical technique

Surgery was performed as previously reported [12, 13]. In particular, the defects were templated and up to four three-dimensional HYAFF^®^ 11 scaffolds (Hyalofast, Anika Therapeutics Srl, Italy) were fashioned to the defect size and shape. The scaffold was soaked in BMAC and implanted in the defect site. In both groups, the scaffolds were secured to the surrounding cartilage using a polydioxanone suture (PDS II 6-0, Ethicon, Somerville, New Jersey, USA) and sealed with fibrin glue (Tissucol, Baxter Spa, Rome, Italy).

### Second-look arthroscopy and biopsy

A second-look arthroscopy was performed in patients who underwent surgical treatment on the same knee for hardware removal or gave their consent while undergoing a surgical procedure on the contralateral knee. During the procedures, the grafts were inspected and evaluated using the ICRS cartilage repair assessment scoring system (degree of defect fill; graft integration to adjacent normal articular surface; and gross appearance of the graft surface). Biopsy samples were obtained and histological and immunohistochemical analyses for collagen type assessment at the regenerated area were performed. Sections of the specimens were stained with haematoxylin and eosin for general histological analysis and with safranin O for evaluation of proteoglycans. On the basis of this analysis, the type of tissue repair was classified as hyaline-like, fibrocartilage, or mixed tissue. Integration of the graft to adjacent normal articular cartilage was also noted. This study was approved by the local ethics committee, Milan, Italy (Prot. N. 14.12.867 Area IV bis).

### Statistical methods

Descriptive and inferential statistical analyses were performed by an independent statistician. Results on continuous measurements are presented on Mean ± SD, and results on categorical measurements are presented in Number (%). Significance is assessed at 5 % level of significance. Student’s t test (two tailed, independent) was used to find the significance of study parameters on continuous scale between two groups (Inter group analysis) on metric parameters. The Student’s t test (two tailed, dependent) was also used to find the significance of study parameters on continuous scale within each group [31]. To demonstrate a difference in KOOS assessments of 10 points, with an expected standard deviation of 10, a prior power analysis determined a necessary sample size of 16 patients in each experimental group (*α* = 0.05, *β* = 0.2). The statistical software used for the analysis of the data was SAS 9.2, SPSS 15.0, Stata 10.1, MedCalc 9.0.1, Systat 12.0 and R environment ver.2.11.1. Statistical significance was defined as follows: * moderately significant (*P* value: 0.01 < *P* ≤ 0.05); ** strongly significant (*P* value: *P* ≤ 0.01).

## Results

The average size of the lesion was 8.5 ± 5.9 cm^2^ in the study group and 9.8 ± 4.4 cm^2^ in the control group. At final follow-up VAS scores improved from 5.4 ± 1.6 to 0.5 ± 0.8 and from 6.0 ± 1.2 to 1.0 ± 0.9; Tegner scores improved from 2 (range 0–4) to 6 (range 4–8) and from 3 (range 0–6) to 6 (range 3–10); IKDC subjective scores improved from 39.2 ± 16.5 to 82.2 ± 8.9 and 40.8 ± 13.9 to 79.4 ± 14.6 for the study and the control group, respectively (Table 3). KOOS scores also showed improvement in all categories, and the differences were statistically significant (*P* < 0.001) in both the study and the control group.

**Table 3:** Summary of results and comparison of study variables between Control and Study groups

Functional outcome scores	(Study group) patient age >45 years			(Control group) patient age <45 years			Control group Vs. study group		
	Preoperative	2-year follow-up	Final follow-up	Preoperative	2-year follow-up	Final follow-up	Preoperative	Preoperative Vs 2-year follow-up	Preoperative Vs final follow-up
VAS	5.6 ± 1.4	0.7 ± 0.9	0.5 ± 0.8	6 ± 1.2	0.9 ± 1	1 ± 0.9	0.145	0.429	0.046*
Tegner	2 (0–4)	5 (3–7)	6 (4–8)	3 (0–6)	6 (3–10)	6 (3–10)	0.344	0.046*	0.437
IKDC Subjective	39.2 ± 16.5	77 ± 12.9	82.2 ± 8.9	40.8 ± 13.9	82.1 ± 10.8	79.4 ± 14.6	0.742	0.149	0.477
KOOS Pain	59.7 ± 15.1	90.1 ± 8.5	92.5 ± 7.3	52 ± 17.1	88.6 ± 10.1	84.8 ± 16.3	0.136	0.439	0.057
KOOS Symptoms	54.9 ± 11.3	84.3 ± 12.4	89.1 ± 10.1	47.9 ± 16.1	81.3 ± 13.4	82.3 ± 15.2	0.120	0.467	0.078
KOOS ADL	61.5 ± 19.7	89.1 ± 11.2	90.7 ± 9.4	59.7 ± 16.7	87.2 ± 14.1	84.3 ± 17.1	0.751	0.640	0.138
KOOS SRA	33.5 ± 15.5	66 ± 19.6	77.2 ± 15.3	30.2 ± 19.9	79.2 ± 17	76.2 ± 21.6	0.557	0.029*	0.813
KOOS QOL	29.9 ± 17.2	72.3 ± 16.6	82.8 ± 10.2	28.5 ± 14.3	81.9 ± 15.7	79.9 ± 19.5	0.781	0.068	0.476

A subgroup analysis of the study group (>45 years) was also done after categorizing the patients based on size of lesion, number of lesions and presence of malalignment (Tables 4, 5, 6). This analysis revealed that (1) patients with lesions measuring <8 cm^2^ had better IKDC subjective scores (86.8 ± 8.7 compared to 76.5 ± 5.2; *P* < 0.006); (2) patient with single lesion had a better IKDC subjective score (85.7 ± 7.7 compared to 76.8 ± 8.1; *P* < 0.023) and better KOOS ADL scores (94.9 ± 7.4 compared to 84.9 ± 8.9; *P* < 0.010); (3) there is no relevant difference between patients receiving single surgical procedure or multiple surgical procedures. No major adverse reactions or postoperative infections were noted besides two subjects with persistent subchondral bone oedema. Intergroup analysis (Table 3) showed better Tegner (*P* = 0.046) and lower KOOS SRA (*P* = 0.029) compared to the control group only at 2-year follow-up.

**Table 4:** Subgroup A of study group based on lesion size

Functional outcome scores at final follow-up	Lesion <8 cm^2^	Lesion >8 cm^2^	*P* value
VAS	0.4 ± 0.7	0.6 ± 0.9	0.571
Tegner	6 (3–10)	6 (3–10)	0.598
IKDC subjective	86.8 ± 8.7	76.5 ± 5.2	0.006**
KOOS pain	95.1 ± 7.8	89.6 ± 5.2	0.086
KOOS symptoms	90.8 ± 11.1	88.2 ± 9.3	0.583
KOOS ADL	93.1 ± 9.8	88.2 ± 8.4	0.254
KOOS SRA	81.4 ± 14.5	72.9 ± 15.3	0.220
KOOS QOL	86.3 ± 11.4	80 ± 10	0.212

Values are described as mean ± SD except for Tegner that is reported as median and range

*IKDC* International Knee Documentation Committee, *KOOS* Knee Osteoarthritis Outcome Score, *ADL* activities of daily living, *SRA* sports and recreational activities, *QOL* quality of life, *VAS* visual analogue scale

* Moderately significant (*P* value:0.01 < *P* ≤ 0.05); ** Strongly significant (*P* value: *P* ≤ 0.01)

**Table 5:** Subgroup B of study group based on lesion number

Functional outcome scores at final follow-up	Single lesion	Multiple lesions	*P* value
VAS	0.3 ± 0.6	0.8 ± 0.9	0.154
Tegner	6 (3–10)	6 (3–10)	0.807
IKDC Subjective	85.7 ± 7.7	76.8 ± 8.1	0.023*
KOOS Pain	94.6 ± 7.3	89.6 ± 6.3	0.133
KOOS Symptoms	90.3 ± 10.3	88.6 ± 10.5	0.720
KOOS ADL	94.9 ± 7.4	84.9 ± 8.9	0.010*
KOOS SRA	80.8 ± 13.8	72.6 ± 16.5	0.240
KOOS QOL	84.1 ± 10.8	82.5 ± 1	0.760

Values are described as mean ± SD except for Tegner that is reported as median and range

*IKDC* International Knee Documentation Committee, *KOOS* Knee Osteoarthritis Outcome Score, *ADL* activities of daily living, *SRA* sports and recreational activities, *QOL* quality of life, *VAS* visual analogue scale

* Moderately significant (*P* value:0.01 < *P* ≤ 0.05); ** Strongly significant (*P* value: *P* ≤ 0.01)

**Table 6:** Subgroup C of study group based on concomitant procedures

Functional outcome scores at final follow-up	Single	Multiple	*P* value
VAS	0.2 ± 0.4	0.6 ± 0.9	<0.001**
Tegner	6 (3–10)	6 (3–10)	0.607
IKDC Subjective	77.9 ± 10.4	84 ± 7.8	0.175
KOOS Pain	92 ± 5.5	92.9 ± 8	0.778
KOOS Symptoms	90.5 ± 7.8	89.3 ± 11.3	0.779
KOOS ADL	93.2 ± 7.8	89.9 ± 10	0.425
KOOS SRA	78.5 ± 16	77.1 ± 15.3	0.852
KOOS QOL	82.7 ± 8.2	83.8 ± 12.2	0.808

Values are described as mean ± SD except for Tegner that is reported as median and range

*IKDC* International Knee Documentation Committee, *KOOS* Knee Osteoarthritis Outcome Score, *ADL* activities of daily living, *SRA* sports and recreational activities, *QOL* quality of life, *VAS* visual analogue scale

* Moderately significant (*P* value:0.01 < *P* ≤ 0.05); ** Strongly significant (*P* value: *P* ≤ 0.01)

### MRI findings

**Fig. 1:**
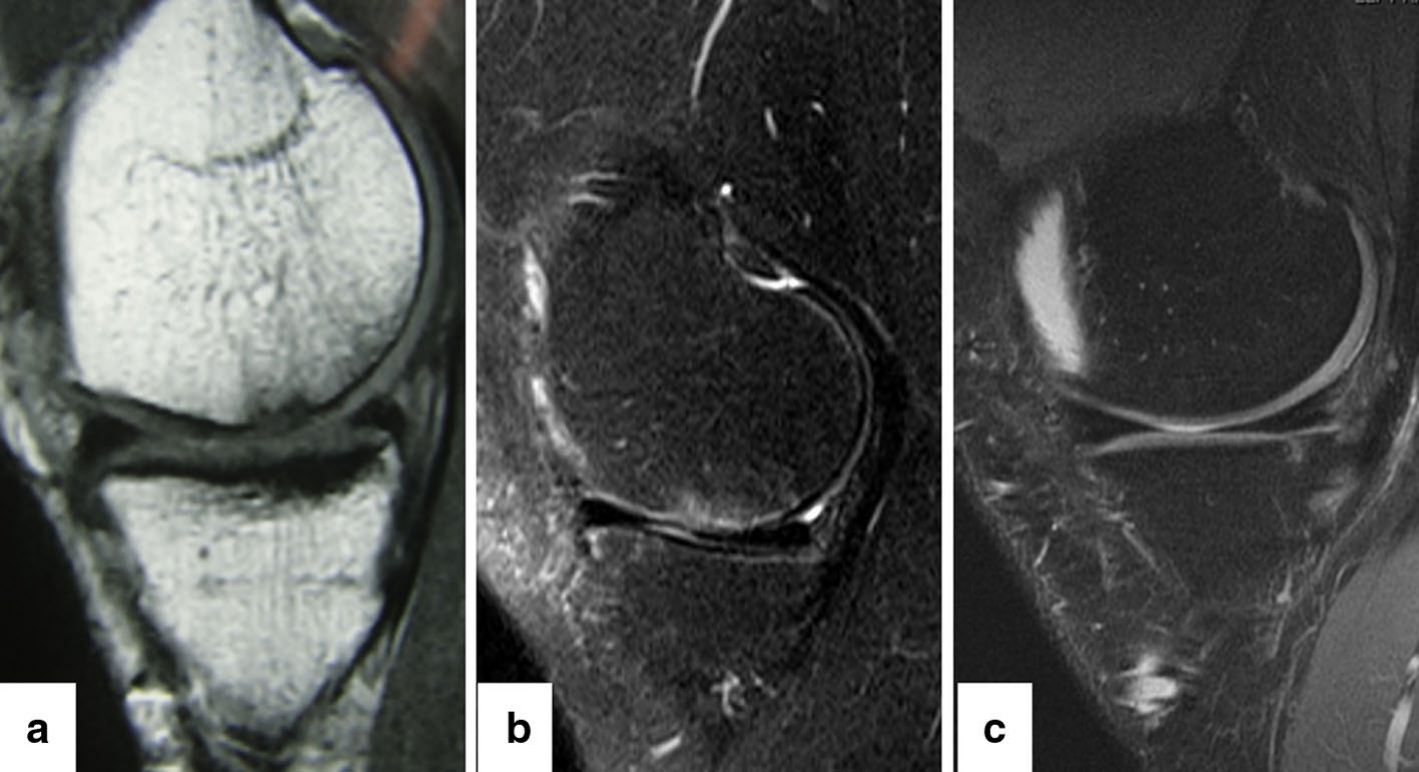
**a** Sagittal section magnetic resonance imaging of a grade 4 chondral lesion involving articular surface of medial femoral condyle in 50-year-old male. **b** 1-year follow-up MRI showing complete filling of the defect. **c** 5-year follow-up MRI showing establishment of smooth articular surface

At final follow-up, MRI findings were available in 16 patients from the study group and 15 patients from the control group (Fig. 1). A complete or near complete (>50 %) filling of the defect was seen in 13 (81 %) patients in the study group and in 10 (71 %) patients in the control group with no signs of hypertrophy. Integration with adjacent cartilage was complete in 15 (93.7 %) patients in the study group and 14 (93 %) in the control group with restoration of the cartilage layer over the subchondral bone. In comparison with the MRI findings at 2 years, no documented deterioration was detected in either group, while the newly formed tissue was still maturing or stabilized at final follow-up.

### Second-look arthroscopy and histological findings

**Fig. 2:**
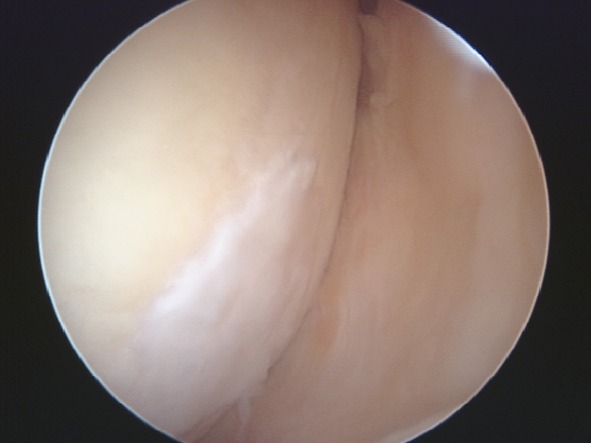
Second look arthroscopy view at 1-year follow-up of grade 4 patellar chondral lesion showing filling of the defect with a well-integrated, smooth surfaced and stable regenerated cartilage

**Fig. 3:**
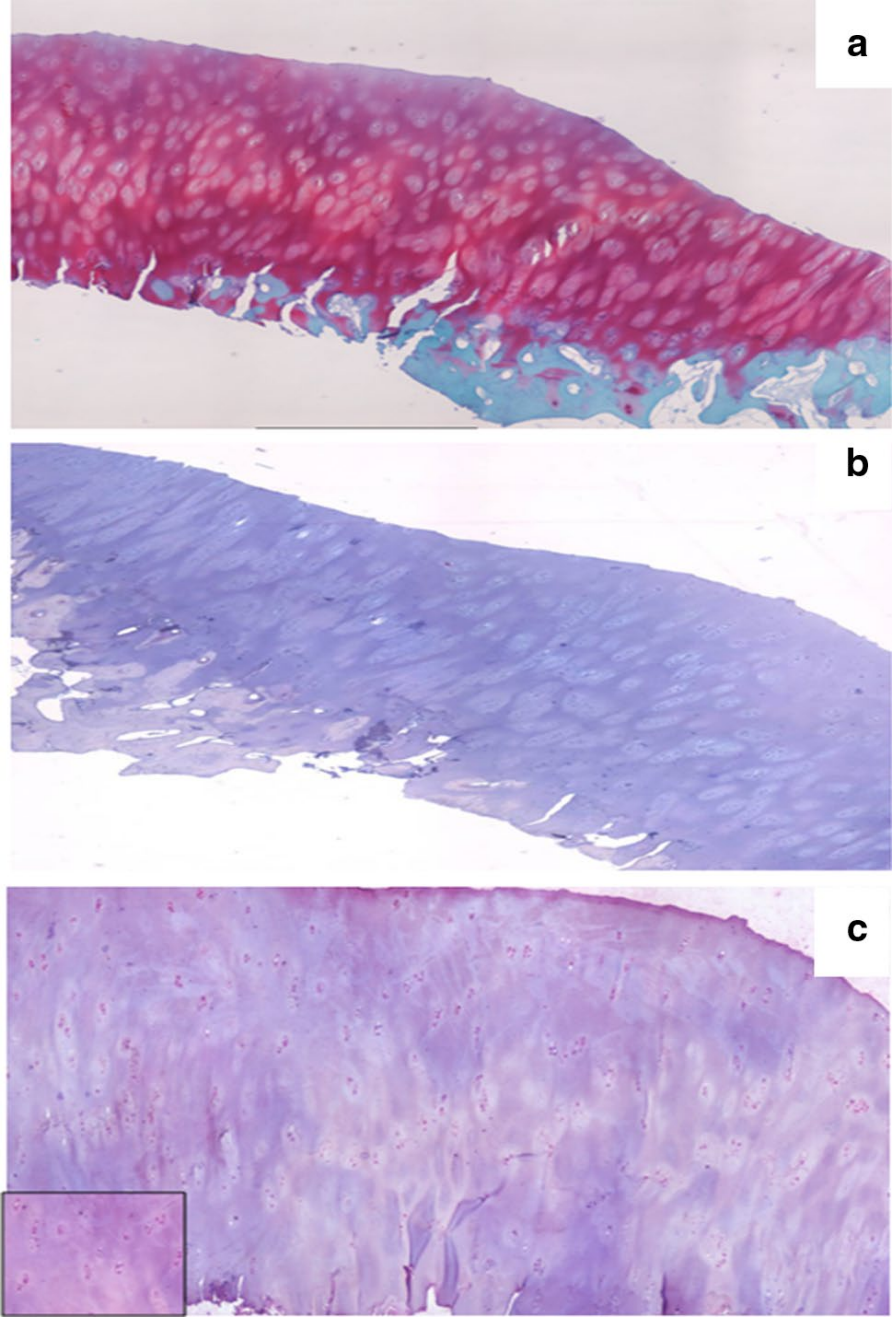
Biopsy report at 2-year follow up. **a** Safranin O staining shows hyaline-like tissue, intensely stained for proteoglycans, slightly hypercellular and with some fibrous features. The superficial layer is regular, the surface is smooth and the cells are homogeneously distributed. The subchondral bone is normal and normal passage bone/cartilage. **b** Collagen type I immunostaining showing no collagen type I positive matrix. **c** Collagen Type II immunostaining showing presence of type II collagen within the matrix

Second-look arthroscopy (Fig. 2) was performed in three patients from the study group and two patients from the control group, at a mean follow-up of 14.4 months. All five patients had concomitant biopsy after obtaining an informed consent (Fig. 3). Results of the second-look arthroscopy and biopsies are summarized in Table 7.

**Table 7:** Second look arthroscopy and histological findings

No.	Location	Lesion size (cm^2^)	Time of second Surgery (Months)	Reason	Histological grading	ICRS cartilage repair assessment score^a^	Overall repair assessment grade^a^
*Study group*							
1	TRO	5	12	Hardware removal (HTO)	Hyaline-like/Fibro cartilage	11	II
2	PAT	6.7	24	Hardware removal (HTO)	Hyaline	12	I
3	PAT	4	12	Contra lateral knee surgery	Mixed (hyaline/fibrocartilage)	11	II
*Control group*							
4	PAT	8	12	Contra lateral knee surgery	Mixed (hyaline/fibrocartilage)	11	II
5	PAT	6.5	12	Hardware removal (HTO)	Fibrocartilage	6	III

^a^Grade I: normal (12), Grade II: nearly normal (11–8), Grade III: abnormal (7–4), Grade IV: severely abnormal (<4)

## Discussion

The most important finding of the present study was that BMAC implantation with Hyalofast is a safe, viable and effective solution for the treatment of full thickness cartilage defects of the knee at medium-term follow-up. In particular, in the >45 years population (1) no significant impairment of results was evident compared to <45 years patients; (2) results were affected by lesion size; (3) results were not affected by concomitant surgical procedures; and (4) results were affected by the number of lesions. According to these results, we believe that contraindication to surgery should not be limited to age but instead focus on concomitant pathologies (e.g. tricompartmental OA, malalignment and instability), comorbidities (e.g. diabetes mellitus, obesity and autoimmune disorders) and other general illnesses. Interestingly, at 2-year follow-up the study group showed better Tegner (*P* = 0.046) and lower KOOS SRA (*P* = 0.029) compared to the control group. This finding depends most likely on the lower physical demands of the aged population that can, therefore, benefit the most from the reduction in pain and improvement in function but with a less intense recreational activity.

A subgroup analysis of the study group showed that patients with a single lesion and/or a lesion measuring <8 cm^2^ showed significantly better results at final follow-up, with a better IKDC subjective score (*P* = 0.023 and *P* = 0.006, respectively). Similar findings were also noticed in our previous reports of this procedure performed in a younger population, with patients having single and smaller lesion showing better outcome scores at short-term follow-up [12, 13]. Taken together, these data suggest that lesion size and number are important predictors of final functional outcomes in both age groups.

At final follow-up, MRI evaluation was not available in all patients, and only five patients were subjected to second look arthroscopy with biopsy and histological examination of the regenerated cartilage. In addition, the extent of cartilage defect filling, integration of the graft, stability of the implant and quality of newly regenerated cartilage were not statistically analysed or correlated with the clinical outcome scores in both the groups. Nevertheless, these radiological and histological evidences of cartilage regeneration complemented and overall confirmed the improvement in clinical outcome scores found at final follow-up.

The treatment of cartilage defects in the >45 years population is a current clinical challenge, as microfractures typically result in high failure rates in older patients [30] and the regenerative potential of articular chondrocytes has been demonstrated to decrease in vitro with age [1]. However, the latter was not confirmed by clinical reports that demonstrated non-inferior results in patients aged >40 years, compared to a matched group of younger patients [26]. Following this experience, we aimed at evaluating the potential, in this particular patient population, of a single-surgery, MSC-based technique for cartilage repair. A review of current literature shows no previous report of MSC-based cartilage regeneration in a population older than 45 years of age. The first clinical study by Wakitani et al. [33] using expanded bone marrow-derived MSCs to repair cartilage defects in OA knees concluded that MSCs were capable of regenerating a functional repair tissue. In a rabbit knee model, Grigolo et al. [16] reported better quality of the regenerated tissue between the implants with scaffolds carrying MSCs compared with the scaffold alone or non-treated lesions in the control group at 6 months. In a comparative prospective study, Nejadnik et al. [25] analysed the clinical outcomes of patients treated with first-generation ACI and those treated with the same procedure but based on MSC injections: at the end of 2 years, patients in both groups had comparable results with lower costs and donor-side morbidity for the MSCs group. Due to the easy availability of autologous bone marrow MSCs and their chondrogenic potential, current research is now focusing on the development of one-step, simple, reproducible and cost-effective procedures to treat cartilage lesions. In this regard, the use of bone marrow aspirate concentrate (BMAC), which contains multipotent stem cells (MSCs) and growth factors, could represent a promising option. Ochi et al. [27] observed that the injection of cultured MSCs combined with microfracture could accelerate the regeneration of cartilage in a rat knee model. An equine study by Wilke et al. [35] showed enhanced chondrogenesis and cartilage healing after arthroscopic implantation of MSCs. Hui et al. [18] compared MSC transplants to cultured chondrocytes, osteochondral autograft and periosteal grafts in animal models of osteochondritis dissecans. Authors found, after performing histological and biomechanical evaluation from the implanted site, that cartilage regeneration with stem cell transplants were comparable to cultured chondrocytes and superior to periosteum and osteochondral autograft in their ability to repair chondral defects [18]. In a nonrandomized prospective study of 15 patients conducted earlier by us using BMAC and Hyalofast, preliminary results showed significant short-term improvement in all the functional evaluation scores. Furthermore, these good outcomes were correlated with MRI, arthroscopy and available biopsy findings [13]. We further investigated and confirmed our preliminary findings in a bigger sample of patients and revealed that this technique provided durable clinical outcomes at medium-term follow-up (average, 41.3 ± 6.7 months) [12]. The present study further supports the use of BMAC and HA scaffold, showing that satisfactory results can be achieved in a broader patient population and that indication to surgery should not be based only on age, but rather on lesion size and number. Limitations of this study include (i) the higher number of concomitant procedures in the study group; (ii) the small number of patients for the sub-analysis; and (iii) the lack of a sound MRI data analysis to be correlated with clinical findings.

An intriguing explanation for these results may come from the new vision of MSC recently proposed by Caplan as “Medicinal Signalling Cells” [6]. According to this concept, MSCs, rather than participating in tissue formation, work as site-regulated “drugstores” in vivo by releasing trophic and immunomodulatory factors and are activated by local injury [6]. We hypothesize that the harvest procedure from the iliac crest may be enough to activate the MSC and allow for the establishment of a regenerative microenvironment within the defect site [6].

## Conclusion

This approach represents a safe, simple, one-step and cost effective technique for the treatment of large, full thickness cartilage defects, also in the >45 years active population. However, a long-term comparative study with a larger sample and with a detailed radiological analysis is desirable in order to ultimately assess the potential of this technique for young and elderly patients.
