# Erratum to: One-step surgery with multipotent stem cells and Hyaluronan-based scaffold for the treatment of full-thickness chondral defects of the knee in patients older than 45 years

**DOI:** 10.1007/s00167-017-4698-0

**Published:** 2017-09-11

**Authors:** Alberto Gobbi, Celeste Scotti, Georgios Karnatzikos, Abhishek Mudhigere, Marc Castro, Giuseppe M. Peretti

**Affiliations:** 1 Orthopaedic Arthroscopic Surgery International (O.A.S.I.) Bioresearch Foundation, Gobbi Onlus, Via GA Amadeo 34, 20133 Milan, Italy; 2 IRCCS Istituto Ortopedico Galeazzi, Milan, Italy; 3 Philippine Orthopeadic Institute, Makati Medical Center, Makati, Philippines; 4 Department of Biomedical Sciences for Health, University of Milan, Milan, Italy

## Erratum to: Knee Surg Sports Traumatol Arthrosc (2017) 25:2494–2501 DOI 10.1007/s00167-016-3984-6

The article “One-step surgery with multipotent stem cells and Hyaluronan-based scaffold for the treatment of full-thickness chondral defects of the knee in patients older than 45 years”, written by “Alberto Gobbi, Celeste Scotti, Georgios Karnatzikos, Abhishek Mudhigere, Marc Castro, Giuseppe M. Peretti”, was originally published Online First without open access. After publication in volume [25], issue [8], page [2494–2501], the author decided to opt for Open Choice and to make the article an open access publication. Therefore, the copyright of the article has been changed to © The Author(s) [2017] and the article is forthwith distributed under the terms of the Creative Commons Attribution 4.0 International License (http://creativecommons.org/licenses/by/4.0/), which permits use, duplication, adaptation, distribution, and reproduction in any medium or format, as long as you give appropriate credit to the original author(s) and the source, provide a link to the Creative Commons license, and indicate if changes were made. The original article was corrected.

